# Factors contributing to COVID-19 vaccine hesitancy in Veterans later in the pandemic

**DOI:** 10.1186/s12889-025-25716-3

**Published:** 2025-12-19

**Authors:** Jeffrey M. Pyne, Karen H. Seal, Jennifer K. Manuel, Adam N. Kaplan, Natalie Purcell, Dan Bertenthal, Denise Esserman, Marie Mesidor, Beth M. DeRonne, Karen Anderson Oliver, Brandon J. Griffin, Hannah Simpson, Nicole Woodruff, Brian Borsari, Leah Tobey-Moore, Hajra Usman

**Affiliations:** 1 Center for Mental Healthcare and Outcomes Research, Central Arkansas Veterans Healthcare System, 2200 Fort Roots Drive, North Little Rock, AR 72114 USA; 2 South Central Mental Illness Research Education and Clinical Center, Central Arkansas Veterans Healthcare System, 2200 Fort Roots Drive, North Little Rock, AR USA; 3 Department of Psychiatry, University of Arkansas for Medical Sciences, Little Rock, AR USA; 4 Integrative Health Service, San Francisco VA Healthcare System, 4150 Clement Street, San Francisco, CA USA; 5 Departments of Medicine and Psychiatry, University of California San Francisco, San Francisco, CA USA; 6 Department of Psychiatry and Behavioral Sciences, University of California, 675 18th Street, San Francisco, San Francisco, CA USA; 7 Minneapolis VA Health Care System, One Veterans Drive, Minneapolis, MN USA; 8 Center for Care Delivery and Outcomes Research, 5445 Minnehaha Avenue South, Building 9, Minneapolis, MN USA; 9 Center for Data to Discovery and Delivery Innovation, San Francisco VA Health Care System, 4150 Clement Street, San Francisco, CA USA; 10 Department of Social and Behavioral Sciences, University of California, San Francisco, CA USA; 11 Department of Biostatistics, Yale School of Public Health, New Haven, CT USA; 12 Northern California Institute for Research and Education, San Francisco VA Health Care System, 4150 Clement Street, San Francisco, CA USA

**Keywords:** Vaccination hesitancy, Veterans, COVID-19 vaccines

## Abstract

**Background:**

Vaccine hesitancy remains a significant public health concern despite the widespread availability of vaccines. Little is known about factors influencing hesitancy among U.S. military Veterans later in the pandemic. The goal of this study was to identify factors contributing to COVID-19 vaccine acceptance and hesitancy among U.S. military Veterans more than one year after vaccine availability.

**Methods:**

This study was part of the COVID-19 Vaccine Acceptance Study (CoVAcS) conducted across ten Veterans Affairs Health Care Systems from February 2022 to April 2023. CoVAcS delivered motivational interviewing-informed training to healthcare providers and staff. Unvaccinated (*N* = 83) and recently vaccinated (*N* = 140) Veterans were surveyed using items from the Centers for Disease Control and Prevention Vaccine Confidence Survey. Univariable and multivariable logistic regression analyses were conducted.

**Results:**

Compared to recently vaccinated Veterans in univariable analyses, unvaccinated Veterans were older, more likely female, reported higher household income, more likely from a rural residence, did not receive recent seasonal influenza vaccine, and were less likely to screen positive for anxiety or depression. Univariable results also demonstrated high levels of trust in VA healthcare providers and staff and in the VA healthcare system among unvaccinated and recently vaccinated Veterans. In addition, 67.5% of unvaccinated Veterans reported not having enough information about COVID vaccines. In multivariable analyses, unvaccinated Veterans were more likely to be female and live in a rural area. They were also less likely to believe the COVID-19 vaccine is safe, trust the seasonal flu vaccine, or screen positive for anxiety compared to recently vaccinated Veterans.

**Conclusions:**

Results from this study suggest that demographic factors associated with Veteran vaccine hesitancy should be assessed over time to increase provider awareness and focus patient communication and outreach efforts. Unvaccinated Veterans indicated a need for more information about the COVID-19 vaccine. Autonomy-supportive and collaborative communication strategies such as motivational interviewing may help providers better understand patient perspectives on vaccine safety, as well as the barriers and potential benefits to vaccination.

**Trial registration:**

ClinicalTrials.gov, TRN: NCT05027464, Registration Date: 30 August 2021.

## Introduction

Over one million people have died from COVID-19 in the United States (U.S.) and over seven million have died from COVID-19 worldwide [1]. On December 11, 2020, the US government authorized emergency use of the first COVID-19 vaccine. Three days later, the Department of Veterans Affairs (VA) administered the first COVID-19 vaccine to a VA patient. Estimates of COVID-19 deaths prevented by vaccination worldwide range from 14.4 to 19.8 million in the first year of COVID-19 vaccine availability [2].

Despite the wide availability of safe and effective COVID-19 vaccines, nearly one-third of the U.S. population [3] and the U.S. military Veteran population [4] remain not fully vaccinated. Vaccine hesitancy, while growing in the U.S., is not a new phenomenon. Prior to the COVID-19 pandemic, the World Health Organization (WHO) named vaccine hesitancy as one of the top ten global threats in 2019 [5]. The WHO defined vaccine hesitancy as the reluctance or refusal to vaccinate despite the availability of vaccines. Factors associated with COVID-19 vaccine hesitancy among Veterans early in the pandemic included younger age; non-Hispanic White ethnicity; living in rural areas; living in a southern state; being single; prior COVID-19 infection; housing and/or food insecurity; having fewer chronic health conditions, not being assigned to a primary care team, distrust in government institutions, and an opportunity to express of autonomy about vaccines not available during time in military service [4, 6–9]. Other documented reasons for general population COVID-19 vaccine hesitancy included concerns about side effects, safety, and efficacy; lack of trust in government to ensure vaccine safety and effectiveness; the rush to approve the COVID vaccines [10]; the pause on the Janssen vaccine for a safety review [11]; lower perceived seriousness of COVID-19 [12]; right-wing political ideology [12]; and pervasive internet and social media misinformation [13].

This paper reports the results of a survey conducted later in the pandemic to examine factors associated with COVID-19 vaccine acceptance among Veterans receiving care through the VA. The Veteran survey responses reported here are part of a larger VA-funded COVID-19 Vaccine Acceptance Study (CoVAcS). The goal of the survey was to identify predictors of COVID-19 vaccine acceptance and hesitancy among Veterans later in the pandemic. For purposes of this study, later in the pandemic was defined as more than one year after the COVID-19 vaccines were available. This timeframe is important because factors associated with COVID-19 vaccine acceptance later in the pandemic may differ from those identified earlier in the pandemic.

## Materials and methods

### Parent study

The CoVAcS study was conducted across two Veterans Healthcare Administration (VHA) regions located in the South-Central and West Coast. Launched in February 2022, CoVAcS was a one-year pragmatic, cluster randomized controlled trial of a Motivational Interviewing (MI)-informed Vaccine Communication Educational Intervention (VCI) for VA providers and staff versus usual vaccine promotional activities in VA [14]. A total of ten Veterans Affairs Health Care Systems (VAHCS), which included their respective medical centers and community-based outpatient clinics (CBOCs), participated in this study. The duration of the trial was from February 2022 – April 2023. A toolkit entitled: “Vaccine Acceptance Communication and Implementation Toolkit: Applying Motivational Interviewing Skills” provides more information about the VCI intervention [15].

### Subjects

 At the start of this study, 36% of Veterans enrolled in VA had not completed primary COVID-19 vaccination [8]. Veterans who received their most current dose of the COVID-19 primary series vaccination after the start of the study and Veterans who remained unvaccinated were recruited to complete a telephone survey. Vaccination status was ascertained using VA administrative data and confirmed by Veteran self-report. All procedures were performed in compliance with relevant laws and institutional guidelines and were approved by the appropriate institutional committees on August 29, 2021 (reference #1629257-3). Informed consent was obtained for experimentation with human subjects and the privacy rights of human subjects were always observed.

### Recruitment

 During the trial period, we used VA administrative data to identify a sociodemographically diverse, purposive sample of Veterans distributed equally across the mid-South and West Coast VA health systems. Veterans were eligible if they had at least one inpatient or outpatient encounter at their site after the trial began and had either not initiated COVID-19 vaccination or had received their most recent dose of the COVID-19 vaccine primary series within the past 150 days, following the start of clinician and staff training at their site. Purposive sampling further prioritized female Veterans (at least 20%), members of racial (35%) or ethnic (15%) minority groups, rural residents (40%), and Veterans younger than 50 years of age (30%). Enrollment reports were generated monthly to monitor the sociodemographic composition and vaccination status of our enrolled sample, helping us meet recruitment targets.

For recruitment, we used an opt-out letter approach by randomly selecting Veterans within each of the prioritized groups. A total of 2,535 opt-out letters were mailed; 371 Veterans were screened, 238 consented, and 223 completed surveys. Of those, 140 were recently vaccinated (62.8%), and 83 (37.2%) were unvaccinated. The first survey was completed in May 2022, and the last survey was completed in August 2023. Given that December 11, 2020, was the date when the first VA patient received a COVID-19 vaccine, all participants in this study were unvaccinated for over one year after authorization and availability of the COVID-19 vaccine.

### Measures

**Fig. 1:**
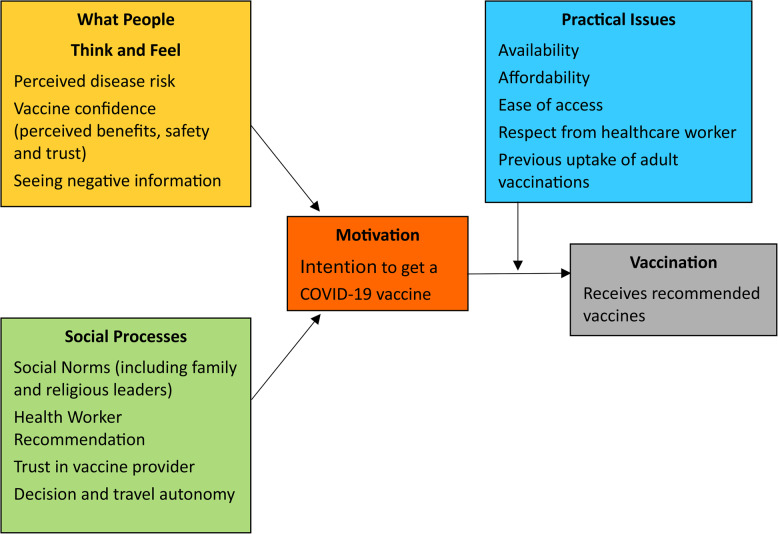
Behavioral and Social Drivers of Vaccination (BeSD) model

 To better understand vaccine hesitancy among Veterans later in the pandemic – at least one year after vaccine availability – we used 21 questions from the CDC Vaccine Confidence Survey Question Bank [16]. This survey tool was informed by the World Health Organization’s Behavioral and Social Drivers of Vaccination (BeSD) framework for COVID-19 vaccination [17] and the work of Brewer et al. [18]. The BeSD includes four domains: What People Think and Feel, Social Processes, Motivation, and Practical Issues (Fig. 1). Other survey items (see Table 1) included sociodemographics, seasonal flu vaccine acceptance, VA primary care experience [19], general health perceptions (overall and mental or emotional health) [20], 5-item primary care post-traumatic stress disorder (PTSD) screener [21], 4-item Patient Health Questionnaire (PHQ) depression and anxiety screener (2 items assessing depression and 2 items assessing anxiety) [22], 3-item Alcohol Use Disorders Identification Test – Consumption (AUDIT-C) [23], and sociodemographics. Additional measures included a 5-item COVID Impacts scale [24], 4 questions about current access to housing and food from the Assessing Circumstances and Offering Resources for Needs (ACORN) survey [25], COVID-19 questions from the 2021 VA Survey of Healthcare Experiences of Patients (SHEP) [19], and the 7-item Discrimination in Medical Settings scale [26].

**Table 1:** – Self-report descriptive sociodemographic and clinical comparisons of recently vaccinated and unvaccinated Veterans^a^

Characteristic	Recently Vaccinated (*N* = 140)	Unvaccinated (*N* = 83)	*p*-value
Age mean (SD)	48.3 (13.7)	55.2 (13.7)	< 0.001
Gender N (%)			0.006
Male	102 (72.9)	44 (53.0)	
Female	37 (26.4)	39 (47.0)	
Ethnicity			0.186
Hispanic or Latino/a/x	24 (17.1)	8 (9.6)	
Not Hispanic or Latino/a/x	114 (81.4)	73 (88.0)	
Race			0.867
American Indian or Alaska Native	3 (2.1)	2 (2.4)	
Asian	2 (1.4)	1 (1.2)	
Black/African American	34 (24.3)	19 (22.9)	
Multiracial	24 (17.1)	9 (10.8)	
Native Hawaiian/Other Pacific Islander	2 (1.4)	1 (1.2)	
White	69 (49.3)	46 (55.4)	
Sexual Orientation			0.153
LGBTQ+	16 (11.4)	4 (4.8)	
Not LGBTQ+	124 (88.6)	79 (95.2)	
Education			0.484
High School (GED) or less	15 (10.7)	11 (13.3)	
Some College or Trade School	90 (64.3)	47 (56.6)	
Bachelor’s degree or more	34 (24.3)	25 (30.1)	
Political Party Affiliation			0.328
Republican	37 (26.4)	31 (37.3)	
Democrat	18 (12.9)	7 (8.4)	
Independent	35 (25.0)	22 (26.5)	
Undecided	29 (20.7)	11 (13.3)	
Rather not say, Don’t know, Other	21 (15.0)	12 (14.5)	
Household Income			0.03
<$ 50 K per year	76 (54.3)	33 (39.8)	
>$ 50 K per year	53 (37.9)	45 (54.2)	
Residence (RUCA Code)			0.002
Rural	21 (15.0)	28 (33.7)	
Urban	119 (85.0)	55 (66.3)	
Flu Vaccine This Season			0.023
Yes	57 (40.7)	19 (22.9)	
No	81 (57.9)	63 (75.9)	
Total PCL-5 Score mean (SD)	2.27 (2.03)	2.39 (1.96)	0.678
PHQ-4 Anxiety Screener			0.039
Yes	66 (47.1)	27 (32.5)	
No	71 (50.7)	55 (66.3)	
PHQ-4 Depression Screener			0.028
Yes	56 (40.0)	21 (25.3)	
No	78 (55.7)	60 (72.3)	
AUDIT-C Total Score mean (SD)	1.94 (2.83)	1.57 (2.27)	0.287
Overall Health			0.379
Excellent	8 (5.7)	9 (10.8)	
Very Good	37 (26.4)	15 (18.1)	
Good	54 (38.6)	35 (42.2)	
Fair	31 (22.1)	18 (21.7)	
Poor	10 (7.1)	5 (6.0)	
Overall Mental or Emotional Health			0.187
Excellent	10 (7.1)	14 (16.9)	
Very Good	29 (20.7)	14 (16.9)	
Good	40 (28.6)	28 (33.7)	
Fair	44 (31.4)	20 (24.1)	
Poor	16 (11.4)	6 (7.2)	

a. N (%) unless otherwise noted. Univariate differences were assessed for significance using Chi-Square tests for proportions and two-sample means tests for continuous outcomes, and p-values were reported. LGBTQ + stands for lesbian, gay, bisexual, transgender, queer/questioning plus non-cisgender and non-straight identities. GED stands for general educational development. PHQ stands for patient health questionnaire. AUDIT-C stands for alcohol use identification test-concise

### Data analysis

 Sociodemographics and clinical variables are descriptively summarized using means (standard deviations) and frequencies (percents) according to vaccination status. Univariable differences were assessed for significance using Chi-Square tests for proportions and two-sample means tests for continuous outcomes with corresponding p-values.

 The sociodemographic and clinical variables in Table 1 and the other survey variables described in the Measures section above were used in the multivariable model building process. The clinical variables (depression, anxiety, and at-risk alcohol use) were added because of their potential relationship to the BeSD domains (e.g., What People Think and Feel, Social Processes, and Motivation). Next, we built a multivariable vaccine acceptance model using the following model building process. First, we screened all survey items both graphically and statistically to assess whether each item’s response pattern differed by vaccination status, using stacked bar charts for visual comparison. We then used Bayesian Effect Fusion (BEF) for logistic regression (0 = unvaccintated, 1 = recently vaccinated) to assess whether responses to selected survey items could be collapsed based on whether responses to the same survey item yielded similar associations with vaccination status [27]. Bayesian ‘significance’ was determined by the 95% Credible Interval (CrI) excluding the null value of one. From this procedure, only the survey questions whose non-reference response levels were not combined to their corresponding reference level were carried forward in the model building. Next, we fit a stepwise logistic regression model, with vaccination status as the dependent variable and the retained survey questions and contextual factors identified above as independent variables. We applied a both-directions stepwise procedure using the lower Akaike Information Criterion (AIC) to further simplify the model and identify better model fit [28]. Results from the stepwise regression are displayed as odds ratios (OR) with corresponding 95% confidence intervals (Table 6). The final multivariable model included 164 Veterans because ambiguous responses (e.g. ‘prefer not to answer’, ‘don’t know’, or no response recorded) were treated as missing (total excluded from analysis, *N* = 59) to make interpretation of results more straight-forward.

## Results

Compared to recently vaccinated Veterans in univariable analyses, unvaccinated Veterans were older, more likely female, reported higher household income, more likely from a rural residence, did not receive recent seasonal influenza vaccine, and were less likely to screen positive for anxiety or depression (Table 1). There were no statistically significant between-group differences in race, ethnicity, sexual orientation, education, political party affiliation, PTSD screening symptoms, risky alcohol use, ratings of overall health or mental/emotional health.

Within the What People Think and Feel domain, overall, there were no significant differences between how recently vaccinated and unvaccinated Veterans responded to questions about the impact of a prior COVID-19 infection on vaccination decision. However, among Veterans who experienced a prior COVID-19 infection, unvaccinated Veterans were more likely than recently vaccinated Veterans to report ongoing COVID symptoms, including feeling tired all the time (34% versus 11.3%; *p* = 0.005), loss of smell or taste (24% versus 9.9%; *p* = 0.064), and headaches (22.0% versus 8.5%; *p* = 0.065).

There were also no significant differences in concerns about the risk of getting COVID or getting it again. However, in response to the question “If you were to get COVID or get it again, how sick do you think you would be?”, recently vaccinated Veterans were more likely to respond, “Very Sick” (12.9%) compared to unvaccinated Veterans (3.6%; overall *p* = 0.043). Unvaccinated Veterans were less likely to perceive the COVID-19 vaccine as safe with 67.5% reporting Not Safe at All (Table 2). There were no significant differences between the two groups in social behaviors to limit risk of COVID-19 infection (e.g., social distancing), perceived rates of COVID-19 infection in social networks, or perceived rate of COVID-19 morbidity or mortality in their social network. However, unvaccinated Veterans were more likely to agree that COVID is man-made (*p* = 0.001) and people are not being told the truth about the safety of the COVID-19 vaccine (*p* < 0.001) or how well the vaccine works (*p* = 0.003).

**Table 2:** How safe do you think the COVID vaccine was or would be for you?

	Recently Vaccinated Veterans (*N* = 140) *N* (%)	Unvaccinated Veterans (*N* = 83) *N* (%)
Very safe	59 (42.1)	1 (1.2)
Moderately safe	39 (27.9)	9 (10.8)
A little safe	19 (13.6)	10 (12.0)
Not at all safe	9 (6.4)	56 (67.5)
I do not know	11 (7.9)	6 (7.2)
Missing	3 (2.1)	1 (1.2)

Within the Social Processes domain, there were no significant difference between recently vaccinated and unvaccinated Veterans in the percentages of patients who reported recent discussions with a member of the VA healthcare team about the COVID-19 vaccine or perceived discrimination when receiving healthcare services. Both groups talked about the vaccine most often with their primary care providers followed by nurses. However, recently vaccinated Veterans reported more trust in the VA provider or staff who recommended the COVID-19 vaccine than unvaccinated Veterans (overall *p* = 0.03) and reported more trust in public health agencies (e.g., CDC or National Institutes of Health) (overall *p* < 0.001). Most unvaccinated Veterans (67.5%) reported trusting the VA provider or staff who recommended the COVID-19 vaccine moderately to very much versus 20.5% reporting similar levels of trust for public health agencies. There was not a statistically significant difference in levels of trust in the VA healthcare system (recently vaccinated versus unvaccinated). Both unvaccinated and recently vaccinated Veterans reported high levels of trust in the VA healthcare system (moderately to very much) 81.9% to 72.1%.

Among 10 potential information sources for learning more about COVID vaccines, there were no significant differences between groups in 7 out of 10 sources (e.g., social media, flyers or brief information sheets, national or local government officials, emails, television or radio news networks, family or friends). There were two sources endorsed more by vaccinated vs. unvaccinated Veterans, respectively: official government website such as VA and CDC (34.3% versus 19.3%; *p* = 0.025) and state or local health departments (24.3% versus 12.0%; *p* = 0.041). One source was endorsed more by unvaccinated Veterans: hearing from or reading about people like me (13.3% versus 5.0%; *p* = 0.053).

Responses to the question about what motivated you the most to get vaccinated (recently vaccinated) or would motivate you if you were to get vaccinated (unvaccinated) are presented in Table 3. One-third of recently vaccinated Veterans (33.6%) reported protecting their own health or the health of family and friends motivated them the most to get vaccinated. Most unvaccinated Veterans (51.8%) reported that nothing would motivate them or that they were unsure what would motivate them. When unvaccinated Veterans were asked if they intended to get vaccinated, most responded “Definitely Not” (80.7%). None of the recently vaccinated or unvaccinated Veterans endorsed increased COVID cases, local hospitals and ICUs filling up with COVID patients, or protecting the health of coworkers as a primary motivator for getting vaccinated. Other primary motivators not endorsed by unvaccinated Veterans included protecting the health of my community, resuming social activities with family and friends, the VA or VA healthcare provider recommending it, family and friends encouraging me, or people thinking less of me. Even recently vaccinated Veterans rarely reported that a VA provider or the VA’s recommendation was their primary motivator for getting the COVID-19 vaccine.

**Table 3:** What motivated you or would motivate you the most to get vaccinated?

Recently Vaccinated Veterans (*N* = 140)	*N* (%)	Unvaccinated Veterans (*N* = 83)	*N* (%)
Protect my health	29 (20.7)	Nothing	36 (43.4)
Protect my family/friends	18 (12.9)	Not sure	7 (8.4)
Get back to work/school	17 (12.1)	Protect my family/friends	7 (8.4)
Resume travel	17 (12.1)	Resume travel	7 (8.4)
Employer required it	17 (12.1)	Enough time has passed for safety	6 (7.2)
Resume social activities	6 (4.3)	Employer required it	5 (6.0)
Participate activities that require it	6 (4.3)	Protect my health	4 (4.8)
VA provider recommended	5 (3.6)	Financial incentive	3 (3.6)
VA recommended	4 (2.9)	Get back to work/school	1 (1.2)
Protect health of my community	1 (0.7)	Participate activities that require it	1 (1.2)
FDA issued full approval	1 (0.7)	Someone I know ill or died of COVID	1 (1.2)
Someone I know ill or died of COVID	1 (0.7)	Protect health of my community	0
Family and friends encouraged me	1 (0.7)	Protect health of my coworkers	0
Not sure	1 (0.7)	Resume social activities	0
Protect health of my coworkers	0	VA provider recommended	0
Enough time has passed for sa16fety	0	VA recommended	0
Financial incentive	0	Family and friends encouraged me	0
Other	16 (11.4)	Other	5 (6.0)

Within the Practical Issues domain, there were no significant differences between recently vaccinated (84.3%) and unvaccinated Veterans (89.2%) reporting that it was or would be Very Easy to get a COVID-19 vaccine, respectively. When asked to respond Yes or No to a list of 13 individual logistic barriers (e.g., cost, too busy, convenience, transportation), very few unvaccinated Veterans (*N* = 0 to 2) or recently vaccinated Veterans (*N* = 0 to 5) endorsed any logistic barriers. There were no differences between recently vaccinated and unvaccinated Veterans in terms of how much the COVID-19 pandemic impacted their life in terms of getting healthcare, meeting basic needs, finances, social support, and mental and emotional health.

Table 4 (unvaccinated Veterans) and Table 5 (recently vaccinated Veterans) show endorsed barriers when asked to either agree or disagree with individual potential barriers to COVID-19 primary series or booster vaccination, respectively. In decreasing order of frequency, unvaccinated Veterans endorsed the following barrier categories: high perceived risk of the vaccine, low perceived risk of COVID infection, and insufficient information about COVID vaccines (67–89%). Individual barriers related to personal beliefs were reported less frequently, including being against religious or philosophical beliefs (22.9%), not believing in vaccines in general (22.9%), and being against political views (12.0%). The COVID-19 booster vaccination barriers reported by recently vaccinated Veterans eligible to receive boosters were similar to unvaccinated Veterans, the most common barriers were related to high perceived risk of the vaccine and low perceived risk of COVID infection and low perceived effectiveness of vaccine (30–32%).

**Table 4:** Unvaccinated veterans’ endorsed barriers to primary series COVID-19 vaccination (*N* = 83)

	*N* (%)
I am concerned the vaccine will make me sick after getting it	74 (89.2)
The vaccines were rushed out too quickly	73 (88.0)
The vaccines have long-term side effects	63 (75.9)
I think the vaccines are more dangerous than getting COVID	61 (73.5)
Most people recover from getting COVID	60 (72.3)
I’m healthy with a strong immune system	59 (71.1)
I am not worried about getting COVID	57 (68.7)
I don’t have enough information about the COVID vaccines	56 (67.5)
I am waiting to see if natural immunity from COVID infection is good enough	55 (66.3)
I do not think COVID is as big a threat as they say	41 (49.4)
I do not need a vaccination because I had COVID and have antibodies	39 (47.0)
The vaccines do not work very well	38 (45.8)
I cannot get a vaccination for health and medical reasons	22 (26.5)
It’s against my religious or philosophical beliefs	19 (22.9)
I do not believe in vaccines in general	19 (22.9)
None of my friends are getting the vaccine	15 (18.1)
I am concerned about vaccine safety related to fertility, pregnancy and/or breastfeeding	15 (18.1)
My doctor hasn’t recommended the vaccine	14 (16.9)
I don’t like needles	14 (16.9)
It’s against my political views	10 (12.0)

Over 95% of the total sample identified their primary care provider as a VA provider in a VA facility. Responses to general questions about their VA primary care provider and general VA healthcare experience demonstrated no significant differences between recently vaccinated and unvaccinated Veterans in questions about trust, communication, and medical skill. Veterans who were recently vaccinated with COVID-19 vaccine were more likely to get the current flu vaccine than unvaccinated Veterans (40.7% versus 22.9%; *p* = 0.023). Trust for the seasonal flu vaccine was also higher among recently vaccinated than unvaccinated Veterans (*p* < 0.001).

Twenty-two survey questions were identified as potential predictors of vaccine uptake and underwent BEF. These questions included beliefs about the COVID vaccine, primary care provider’s relationship with patient, trust in VA care, and access to COVID vaccine. Two BeSD survey questions (vaccine safety and being told truth about vaccine) and 12 sociodemographic and clinical items were identified as candidate predictors after the BEF step. The full model with 14 predictors yielded an AIC of 122.46. After applying bi-directional stepwise regression, the resulting final model with 7 predictors had an AIC of 113.11, suggesting improved model fit over the saturated model (Table 6). In the final model, we found that recently vaccinated Veterans were more likely to perceive the COVID-19 vaccine as at least a little safe, more likely to trust the seasonal flu vaccine at least a little, male, live in urban areas, and more likely to screen positive for anxiety.

## Discussion

Vaccine hesitancy is increasing and remains a potential public health threat. Univariable results from nationwide samples of Veterans during an earlier phase of the COVID-19 pandemic identified unvaccinated Veterans as younger, male, White, living in a rural area, and not getting the recent seasonal flu vaccine [4, 6, 29]. In our late-pandemic study, unvaccinated Veterans were more likely to be older, female, living in a rural area, not getting the recent seasonal flu vaccine, with no differences in vaccination rates by race or ethnicity. In multivariable analysis in our study, female Veterans and living in a rural area were significant sociodemographic predictors of unvaccinated Veterans. Sociodemographic predictors of unvaccinated Veterans that were consistent between prior studies (conducted early in the pandemic) and our study (conducted later in the pandemic) were rural status and not receiving the recent seasonal flu vaccine. Taken together, these results suggest that it may be important to track sociodemographic differences throughout the course of a pandemic to keep clinician and staff awareness current and public health messaging consistent with shifting trends in vaccine hesitancy.

In general, there were few statistically significant univariable differences in BeSD items between recently vaccinated and unvaccinated Veterans’ responses. For example, there were no statistically significant behavioral and social differences between recently vaccinated and unvaccinated Veterans in the BeSD Practical Issues domain items. This suggests that logistical barriers to getting the COVID-19 vaccine later in the pandemic (e.g., cost, time constraints, transportation, inconvenient hours, wait time) and food/housing instability were either addressed by VHA and/or other policies or otherwise did not influence vaccine receipt [30, 31].

Questions from the BeSD Social Processes domain suggested that unvaccinated Veterans were less likely to trust VA providers and staff and public health agencies as sources for information about COVID-19 vaccinations than recently vaccinated Veterans. However, most unvaccinated Veterans (67.5%) reported trusting the VA provider or staff who recommended the COVID-19 vaccine moderately to very much. This high level of trust in VA providers and staff coupled with 67.5% of unvaccinated Veterans reporting not having enough information about the COVID vaccines suggests that VA providers and staff may be an ideal source for providing this information. A recent survey of veterans from a single VAHCS found that sources of trust and advice (including VA health care) identified four unique clusters of veterans and COVID-19 vaccination rates varied among these clusters by more than 3-fold [32].

According to a recent Pew Research Center survey (October 2024), public trust in science and scientists is at an all-time low [33]. The VA’s FY18-24 Strategic Plan [34] and the 2018 American Board of Internal Medicine Foundation Forum on [Re]Building Trust [35] both highlight the importance of patient experience and building patient trust. A pre-COVID study found factors correlating with lack of trust in VA providers included interpersonal factors such as providers and staff not caring, not taking problems seriously, not asking for patient’s opinions, and not showing respect [36]. To promote patient/provider trust, Zulman and colleagues identified five categories of evidence-based activities: (1) prepare with intention; (2) listen intently and completely; (3) agree on what matters most; (4) connect with the patient’s story; and (5) explore emotional cues [37]. These categories are consistent with a MI approach and building patient/provider trust and rapport [38].

Unvaccinated Veterans were also more likely than recently vaccinated Veterans to want to learn more about COVID vaccines from people who were like them (13.3% versus 5.0%). Qualitative data from the same sample suggested that “people like me” could be referencing friends, family and peers; interviewed Veterans frequently cited the experiences of friends, family, and peers with vaccination and with COVID-19 disease as reasons for their vaccine acceptance and hesitancy [39]. Taken together, these findings suggest that a peer-to-peer presentation of information may also be useful for addressing the need for more vaccine information. This could be implemented in-person, over the phone, via televideo, and/or online. An effective online resource is the AboutFace program hosted by the VA National Center for PTSD website where brief video testimonials are posted from a variety of Veterans talking about their experiences getting treatment for PTSD [40]. An evaluation of AboutFace found it to be a promising approach and relevant to a wide range of health conditions [41]. Another approach could be a peer referral program similar to what was pilot tested later in the COVID-19 pandemic using referral coupons distributed by a person who was recently vaccinated and was found to be effective and acceptable [42]. A systematic review of six peer-based vaccine education interventions reported promising results but studies with vaccine hesitant groups are needed [43].

What stands out in Table 3 where Veterans were asked to select one motivation for vaccination from the list, 43.4% of unvaccinated Veterans reported nothing would motivate them to get the COVID vaccine. This means that over half of unvaccinated Veterans thought there might be something that would motivate them. Consistent with the MI approach, it may be more effective for providers to explore Veterans’ concerns, beliefs, motivations and goals with approaches that support autonomy and convey empathy rather than simply recommending the vaccine. MI theory and findings suggest that a strong persuasive approach is less likely to lead to positive behavior change/acceptance, when compared to a more engaging, evocative MI-consistent approach [44].

Differences in responses to the Think and Feel domain items suggested that unvaccinated Veterans were more likely to experience post-COVID-19 infection symptoms and at the same time, were less likely to think they would get very sick if they got infected with COVID-19 in the future. This response pattern is consistent with confirmation bias where in this case the decision to remain unvaccinated is supported by the belief that they are less likely to get very sick despite experiencing more COVID-19 symptoms [45]. Similarly, unvaccinated Veterans were more likely to report that the COVID-19 vaccine was less safe and that people were not being told the truth about vaccine safety or how it works, compared to recently vaccinated Veterans – despite presumably similar access to information reporting on the vaccine’s safety and mechanism of action. Similar barriers to COVID-19 vaccination were reported by unvaccinated Veterans in Table 4 (e.g., concerned about vaccine safety risk more than risk of COVID-19 infection) and in multivariable analysis (COVID vaccine safety). These Veteran responses mirrored a general population sample of unvaccinated adults who were interviewed more than one year prior to the results reported here suggesting similar barriers among Veteran and general population samples [46].

**Table 5:** Recently vaccinated veterans’ endorsed barriers to COVID-19 booster vaccination (*N* = 140)

	*N* (%)
I am worried about the side effects	45 (32.1)
I don’t think I will need it	45 (32.1)
I am concerned about its effectiveness	42 (30.0)
I have not received a recommendation from a health care provider	34 (24.3)
I don’t know when I need to get it	23 (16.4)
I don’t know which brand to get	20 (14.3)
It does not have full FDA-approval	17 (12.1)
I had a bad reaction after my previous vaccination	17 (12.1)
I don’t have time to get it	12 (8.6)
I would have to travel too far to get it	12 (8.6)
I cannot take time off from work	4 (2.9)
I am worried about the costs	3 (2.1)
I don’t know where to get it	3 (2.1)
None of the above	16 (11.4)

Interestingly, the barriers for getting the primary series vaccine among unvaccinated Veterans (Table 4) were similar to the barriers reported by recently vaccinated Veterans considering the booster (Table 5), except that a higher proportion of unvaccinated Veterans endorsed each barrier. This result suggests that accepting the primary series does not mean that the same Veteran will accept a booster. In addition, it appears that similar barriers—such as concerns about vaccine safety, low perceived need for vaccine, and questions about vaccine effectiveness—will be encountered for boosters as with the primary series vaccinations.

**Table 6:** Final Stepwise multivariable logistic model predicting COVID-19 vaccination status (*N* = 164)^a^

	Odds Ratio (95% CI)
People are not being told the truth about the safety of the COVID vaccines (Ref: Agree, Strongly Agree) Disagree, Strongly Disagree, Neither Agree or Disagree	3.28 (0.99, 11.61)
How safe do you think a COVID vaccine was/would be for you? (Ref: Not at all safe)	
A Little Safe	**12.00 (3.12**,** 56.00)**
Moderately Safe	**21.00 (5.33**,** 105.08)**
Very Safe	**333.17 (42.00**,** --)**
How much do you trust the seasonal flu vaccine? (Ref: Not at all safe) A little to very much	**3.36 (1.01**,** 12.00)**
Self-Report sex (Ref: Male)	
Female	**0.22 (0.06**,** 0.71)**
Location (Ref: Rural)	
Urban	**4.58 (1.02**,** 24.44)**
PHQ-4 (Ref: Positive Screen)	
Anxiety Screen Negative	**0.20 (0.04**,** 0.81)**
Depression Screen Negative	0.35 (0.08, 1.39)

a. In logistic model 0 = unvaccinated and 1 = recently vaccinated. The following variables were removed from the model in the stepwise process: age, race, education, household income, sexual orientation, political affiliation. *N* = 164 is based on setting ambiguous answers (i.e., prefer not to answer and don’t know) to missing to facilitate interpretation of results. Statistically significant results are bolded. Upper limit of confidence interval “--” signifies infinite

Qualitative interviews with providers and COVID-19 unvaccinated Veterans from this study showed that providers often attributed reasons for vaccine-hesitancy among Veterans that were different from those that Veterans actually endorsed [39]. For example, VA providers felt that misinformation and conspiracy theories drove vaccine-hesitancy, while unvaccinated Veterans more often cited personal experiences and observations as the basis for their concerns about vaccine side-effects and safety. VA providers also felt that high quality information about vaccines was readily accessible; unvaccinated Veterans did not. These perception differences could certainly have a negative impact on effective communication and provider-patient relationships. These qualitative interviews also suggested that unvaccinated Veterans generally wanted more discussion with their VA providers about vaccine research, not less.

Taken together, these results suggest that a MI approach and peer-to-peer communication may improve communication and address other late-pandemic vaccine acceptance barriers. In addition, a MI approach (e.g., use of open-ended questions, reflective listening, understanding motivations/concerns, sharing information with permission, and negotiating next steps) is an effective approach for addressing patient-level confirmation bias [47, 48]. A MI approach can also mitigate provider bias and help providers better understand Veterans’ concerns, motivations, and the experiences that inform their decisions [49]. As mentioned above, more studies using peers in educational efforts and messaging are needed with vaccine hesitant groups. In addition, artificial intelligence (AI) methods are being used to individualize the risk/benefit analysis of vaccination, identify factors influencing vaccine uptake, use social media data to identify barriers, counter misinformation, and deploy personalized communication and outreach, including the pilot testing of an AI-driven chatbot with MI skills to decrease COVID-19 vaccine hesitancy [50, 51]. There are also risks associated with training AI on non-representative datasets that can amplify misinformation, anger, and fear [52]. Therefore, safeguards are needed to provide ethical guidelines and regulations for use in public health including transparency, data privacy, workforce training, and the involvement of the local community and healthcare providers [53].

Strengths of this study included the use of the CDC Vaccine Confidence Survey questions and opt-out letters to randomly select Veterans to achieve a purposive sample. Limitations included a decreasing number of recently vaccinated Veterans to sample from. Also, the Veteran-only sample and the sample being limited to two US geographic regions (Mid-South and Western United States) may limit generalizability to other geographic regions or non-Veteran samples.

## Conclusions

Results from this study suggest that demographic factors associated with Veteran vaccine hesitancy should be assessed over time, and peers and VA providers be involved in sharing vaccine information. Persistently vaccine hesitant Veterans were not swayed in their vaccination decision by their own post-COVID-19 symptoms or the perceived risk of future COVID-19 infection. Unvaccinated Veterans later in the pandemic continued to express concerns about vaccine safety, transparency around how the vaccine works, and whether they were being told the truth about the COVID-19 vaccine. They also indicated a need for more information about the COVID-19 vaccine. Using a MI approach in vaccine conversations may be useful for informing patient decisions about vaccines.

## Data Availability

The datasets used and/or analyzed during the current study are available from the corresponding author on reasonable request.

## Abbreviations

ACORN: Assessing Circumstances and Offering Resources for Needs
AI: Artificial Intelligence
AIC: Akaike Information Criterion
AUDIT-C: Alcohol Use Disorder Identification Test – Consumption
BEF: Bayesian Effect Fusion
BeSD: Behavioral and Social Drivers of Vaccination
CBOC: Community Based Outpatient Clinic
CDC: Center for Disease Control
CI: Credible Interval
CoVAcS: COVID Vaccine Acceptance Study
COVID: Coronavirus Disease of 2019
FY: Fiscal Year
ICU: Intensive Care Unit
MI: Motivational Interviewing
OR: Odds Ratio
PHQ: Patient Health Questionnaire
PTSD: Post Traumatic Stress Disorder
SHEP: Survey of Healthcare Experiences of Patients
US: United States
VA: Veterans Affairs
VAHCS: Veterans Affairs Health Care System
VHA: Veterans Healthcare Administration
WHO: World Health Organization
VCI: Vaccine Communication Educational Intervention
